# Target organs for lymphocystis disease virus replication in gilthead seabream (*Sparus aurata*)

**DOI:** 10.1186/s13567-017-0428-3

**Published:** 2017-04-11

**Authors:** Estefania J. Valverde, Juan J. Borrego, M. Carmen Sarasquete, Juan B. Ortiz-Delgado, Dolores Castro

**Affiliations:** 1 Departamento de Microbiología, Facultad de Ciencias, Universidad de Málaga, Campus Universitario Teatinos, Malaga, Spain; 2 Instituto de Ciencias Marinas de Andalucía, CSIC, Puerto Real, Cádiz Spain

## Abstract

The lymphocystis disease (LCD), the main viral pathology described in cultured gilthead seabream (*Sparus aurata*), is a self-limiting condition characterized by the appearance of hypertrophied fibroblasts (named lymphocysts) in the connective tissue of fish, primarily in the skin and fins. The causative agent of the disease is the Lymphocystis disease virus (LCDV), a member of the *Iridoviridae* family. In the present study, LCDV genome and transcripts were detected by real-time PCR in caudal fin, as well as in several internal organs, such as intestine, liver, spleen, kidney and brain, from asymptomatic, diseased and recovered gilthead seabream juveniles. These results indicate that the LCDV has a broad range tissue tropism, and can establish a systemic infection, even in subclinically infected fish. As showed by in situ hybridization, the permissive cells for LCDV infection seem to be fibroblasts, hepatocytes and cells of the mononuclear phagocyte system. Histopathological alterations associated with LCD were observed in all the organs analysed, including necrotic changes in liver and kidney, inflammatory response in the intestine submucosa or brain haemorrhage, although lymphocysts were only detected in the dermis of the caudal fin. Nevertheless, these histological changes were reverted in recovered animals.

## Introduction

Lymphocystis disease (LCD) is the viral infection most frequently reported in gilthead seabream (*Sparus aurata*) farms in the South Atlantic and Mediterranean areas [1]. The aetiological agent of this disease is the Lymphocystis disease virus (LCDV), a member of the *Iridoviridae* family [2].

LCD is a self-limiting disease characterized by the hypertrophy of fibroblastic cells in the connective tissue of fish [3]. These hypertrophied cells, named lymphocysts or lymphocystis cells, are usually observed on the skin and fins, although they have also been described in several internal organs (such as the stomach, spleen, liver, kidney and heart) [4–8]. In gilthead seabream, lesions associated with LCD have only been observed in the skin and fins of affected fish, and usually disappear after 20–45 days depending on water temperature [9–11].

Data on LCDV pathogenesis are very scarce and generally limited to histopathological studies of skin lesions [9, 12, 13]. However, several studies have shown that viral antigens can be detected in a number of organs of infected fish, not only in lymphocystis lesions [13–15]. In gilthead seabream, DNA–DNA hybridization and immunohistochemistry were used to detect LCDV in diseased and recovered juveniles [14]. Viral genomes and antigens were detected in the different organs analysed, including the caudal fin, gills, intestine, liver, spleen and kidney, suggesting that LCDV establishes a systemic and persistent infection in this fish species. In addition, LCDV is frequently detected by PCR-based methods in apparently healthy seabream [14, 16, 17], which indicates that they may be subclinically infected. However, further studies are necessary to confirm these results and to establish if these infections are productive.

Thus, the objective of the present study was to determine the target organs and cells that support LCDV replication in gilthead seabream juveniles, both lymphocystis (LC)-diseased and subclinically infected. In addition, a histopathological study of LCD was also conducted.

## Materials and methods

### Fish samples

Gilthead seabream specimens were obtained from two fish farms located in southwestern Spain. In the first farm, fish without signs of LCD (6–10 g in weight) were collected, and constituted the group named “asymptomatic”. This farm had no record of any LCD outbreaks in over 15 years. In the second farm, diseased individuals (6–10 g) showing typical external signs of LCD were collected, and 2 months after the disease signs had disappeared, another group of fish from the same population (15–20 g) was sampled. These fish constituted the “diseased” and the “recovered” groups, respectively. Fish used in this study were treated according to the Spanish directive (RD 53/2013, BOE no. 34), and were euthanized by anaesthetic overdose (MS-222, Sigma-Aldrich, St. Louis, MO, USA).

Samples of the caudal fin, intestine, liver, spleen, kidney and brain of nine individuals from each experimental group were aseptically collected and individually processed for subsequent homogenization and nucleic acid extraction. In addition, the same organs were collected from three fish in each group for in situ hybridization (ISH) and histological examination. Fish samples were fixed in 4% paraformaldehyde (Sigma-Aldrich) in DEPC-treated PBS (pH 7.2) for 24 h at 4 °C, and embedded in paraffin using standard histological procedures. Fixed caudal fin samples were decalcified with a solution containing 10% EDTA (Sigma-Aldrich) and 4% paraformaldehyde in DEPC-treated water at pH 7.0 for 10–15 days at 4 °C. Tissue sections (5–7 µm) were mounted on TESPA (3-triethoxysilylpropylamine, Sigma-Aldrich)-treated slides.

### DNA and RNA extraction and cDNA synthesis

Total DNA and RNA were extracted using the E.Z.N.A. Tissue DNA Kit and the E.Z.N.A. Total RNA Kit I (Omega Bio-tek Inc., Norcross, GA, USA), respectively, following the manufacturer’s instructions. Total RNA was treated with RNase-free DNase I (Roche Applied Science, Mannhein, Germany) for 30 min at 37 °C. RNA purity and quantity was determined using NanoDrop 1000 (Thermo Scientific, West Palm Beach, FL, USA). After DNase treatment, total RNA was used in the qPCR reaction in order to control for the absence of viral genomic DNA. First-strand DNA synthesis was carried out with 1 µg of total RNA and random hexamer primers using the Transcriptor First Strand cDNA Synthesis Kit (Roche). DNA and cDNA were stored at −20 °C until used as template for qPCR.

### LCDV DNA quantification and gene expression

The qPCR protocol described by Valverde et al. [17] was used to quantify the amount of viral DNA in the samples. The number of copies of LCDV DNA was calculated by interpolation in a standard curve, and viral loads expressed as viral DNA copies per milligram of tissue.

Relative quantification of major capsid protein (MCP) gene expression was carried out by RT-qPCR, following the protocol mentioned above but using 20-µL final volume reactions and 2 µL of cDNA (at a 1/30 dilution). Normalized relative MCP expression levels were calculated for each sample applying the formula: F = log_10_ [(E + 1)^40−Ct^/N] [18], where E is the amplification efficiency of the qPCR, Ct (threshold cycle) corresponds to the PCR cycle number, N is the maximal number of viral DNA copies/mg of tissue detected minus the number of viral DNA copies/mg of tissue determined by absolute qPCR for the sample, and Ct of 40 arbitrarily corresponds to “no Ct” by qPCR.

Results obtained for viral DNA quantification and relative gene expression were analysed statistically using a Mann–Whitney U test followed by a Holm–Bonferroni correction for multiple comparisons.

### RNA in situ hybridization and histopathology

Digoxigenin (DIG)-labelled RNA probes were synthesized by in vitro transcription with the DIG RNA Labelling Kit (Roche) using a 150-bp fragment of the viral MCP gene (nucleotide positions 173–322 of the LCDV SA9 MCP gene, GenBank accession no. GU320728) cloned into the pCRII Dual Promoter vector (Invitrogen, Life Technologies, Carlsbad, CA, USA) as the template. The RNA probes were produced from 1 µg of linearized plasmid using T7 (antisense) or SP6 (sense) polymerases.

Deparaffinised and rehydrated tissue sections were permeabilized for 30 min with 10 µg/mL proteinase K in a buffer containing Tris–HCl 0.05 M pH 7.6 (40%, v/v) and CaCl_2_ 1 M (4%, v/v) in DEPC-treated water at 37 °C. Sections were pre-hybridized with formamide (50%, v/v), 20× SSC (25%, v/v), torula yeast RNA (50 mg/mL), heparin sodium salt (5 mg/mL), Denhardt’s solution (2%, v/v), CHAPS (2%, w/v) and Tween 20 (0.5%, v/v) in DEPC-treated water for 3 h in a 2× SSC saturated atmosphere at 55 °C. Sense and antisense probes were denatured at 85 °C for 5 min, and hybridization was performed overnight at 60 °C. After hybridization, sections were washed as previously described [19] and treated with 1% blocking reagent (Roche) in maleic acid buffer (0.1 M maleic acid, 0.15 M NaCl, pH 7.5) for 1 h at room temperature. Then, the slides were incubated with anti-digoxigenin-AP (Roche) overnight at 4 °C, and the hybridization signals were detected using NBT/BCIP (Roche) according to the manufacturer’s instructions. All reagents were supplied by Sigma-Aldrich unless otherwise stated. Finally, sections were dehydrated and mounted in Eukitt^®^ quick-hardening mounting medium (Sigma-Aldrich).

In parallel, tissue sections were stained with haematoxylin-eosin (HE) and haematoxylin-V.O.F. [20] for histological examination.

## Results

### Viral load and gene expression

**Figure 1:**
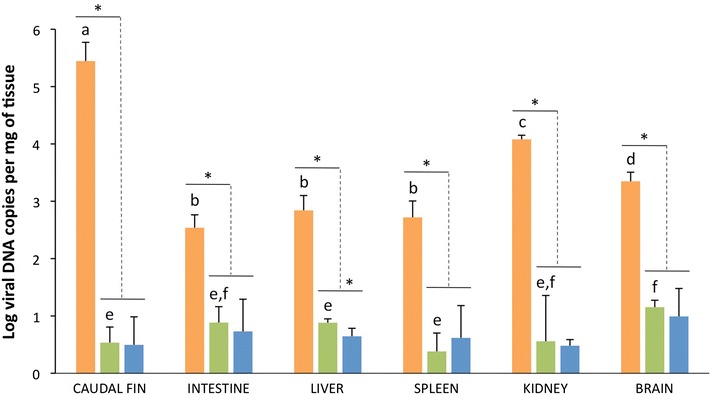
**Viral loads in samples from different organs of gilthead seabream determined by qPCR.** Experimental groups: diseased, orange; asymptomatic, green; recovered, blue. Different letters indicate significant differences between organs in the same experimental group. *Significant differences between groups. Significant level *p* < 0.01 (Mann–Whitney U test, Holm–Bonferroni correction). Error bars represent ± standard deviation (*n* = 9).

LCDV was detected in all the samples analysed by qPCR. The viral load in the different organs examined from the three experimental groups is shown in Figure 1. In diseased fish the highest viral loads were detected in the caudal fin (3.5 ± 2.4 × 10^5^ copies of viral DNA/mg of tissue), followed by the kidney (1.2 ± 0.2 × 10^4^ copies of viral DNA/mg) and brain (2.4 ± 0.9 × 10^3^ copies of viral DNA/mg). In fish from the asymptomatic and recovered groups, low-titre infections were observed, with estimated viral loads between 0.4 and 27.5 copies of viral DNA per mg. No significant differences (*p* < 0.05) were observed between the asymptomatic and the recovered groups, except for liver samples. In fish from the asymptomatic group, the number of LCDV DNA copies in the brain was significantly higher than in the caudal fin (*p* < 0.01).

**Figure 2:**
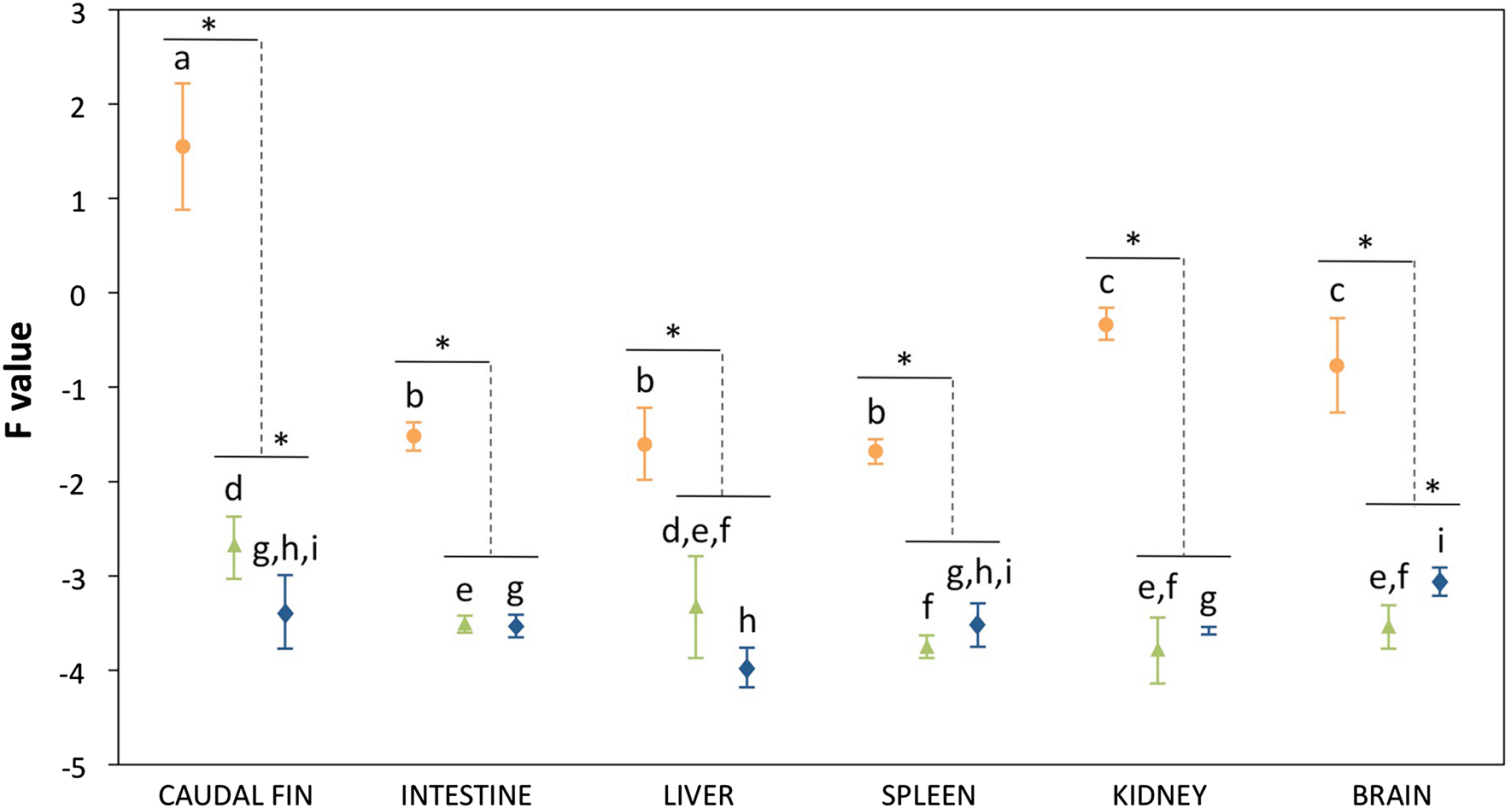
**Relative MCP gene expression values in samples of gilthead seabream juveniles.** Experimental groups: diseased, orange; asymptomatic, green; recovered, blue. Different letters indicate significant differences between organs in the same experimental group. *Significant differences between groups. Significant level *p* < 0.01 (Mann–Whitney U test, Holm–Bonferroni correction). Error bars represent ± standard deviation (*n* = 9).

MCP gene expression was analysed as an indicator of viral productive infection. In diseased fish, relative viral gene expression values were similar to viral loads in the different organs analysed (Figure 2). Thus, the highest relative expression values were detected in the caudal fin, followed by those in the kidney and brain. F values were significantly higher (*p* < 0.01) in these organs than in the other internal organs analysed. Viral gene expression was observed in all organs collected from fish from the asymptomatic and recovered groups, with relative values significantly lower (*p* < 0.01) than those obtained in samples from diseased fish. In asymptomatic fish, F values in the caudal fin were significantly higher (*p* < 0.01) than those in other tested organs, with the exception of the liver. No significant differences (*p* < 0.01) were observed between both experimental groups except for the caudal fin and brain samples, with F values significantly higher in the asymptomatic and recovered groups, respectively.

### RNA in situ hybridization

Viral transcripts were detected by ISH in all organs from the diseased fish, whereas no signal was observed in the sections from fish belonging to the asymptomatic and recovered groups (results not shown). No labelling was observed in the negative controls using sense probe for ISH.

**Figure 3:**
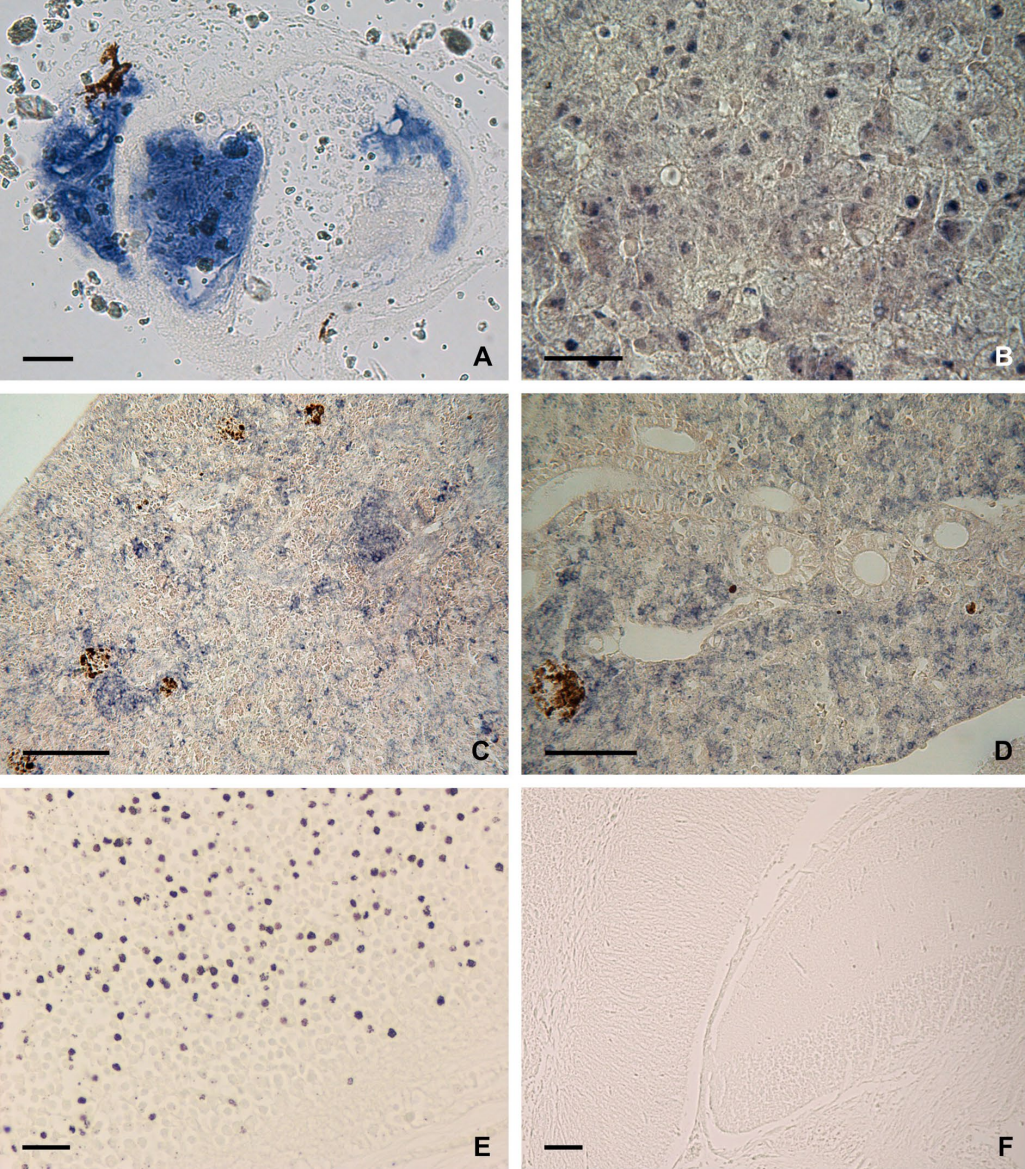
**LCDV detection in tissues from gilthead seabream demonstrated by in situ hybridization (ISH).** The signal is observed microscopically as dark blue staining. **A** Lymphocyst in caudal fin showing viral mRNA in its cytoplasm. **B** ISH-positive hepatocytes in the liver of diseased fish. **C**, **D** Hybridization signal in the splenic pulp and in kidney interstitial cells, respectively, form diseased fish. **E** Viral transcripts in brain section from diseased fish. **F** ISH-negative brain section from recovered fish. Scale bars: (**A**, **B**, **D**) 50 µm; (**C**, **F**) 100 µm; (**E**) 20 µm.

In sections of the caudal fin, the hybridization signal was strong, and labelling was observed as cytoplasmic inclusions in the lymphocysts (Figure 3A) and in some cells in the surrounding connective tissue. In liver sections, numerous hepatocytes showed marked labelling in their cytoplasm (Figure 3B). The hybridization signal was widely distributed in the splenic pulp, although in some areas the signal appeared concentrated around melanomacrophage centres (MMC) and ellipsoids (Figure 3C). In the kidney, the hybridization signal was mostly confined to the haematopoietic tissue (Figure 3D). In sections from the brain, ISH labelling was observed in the cytoplasm of cells in the granular layer (Figure 3E). Finally, tissue sections from the intestine were also ISH-positives, but the tissue was so damaged by the ISH protocol that it was not possible to distinguish the localization of the labelled cells.

### Histopathological study

**Figure 4:**
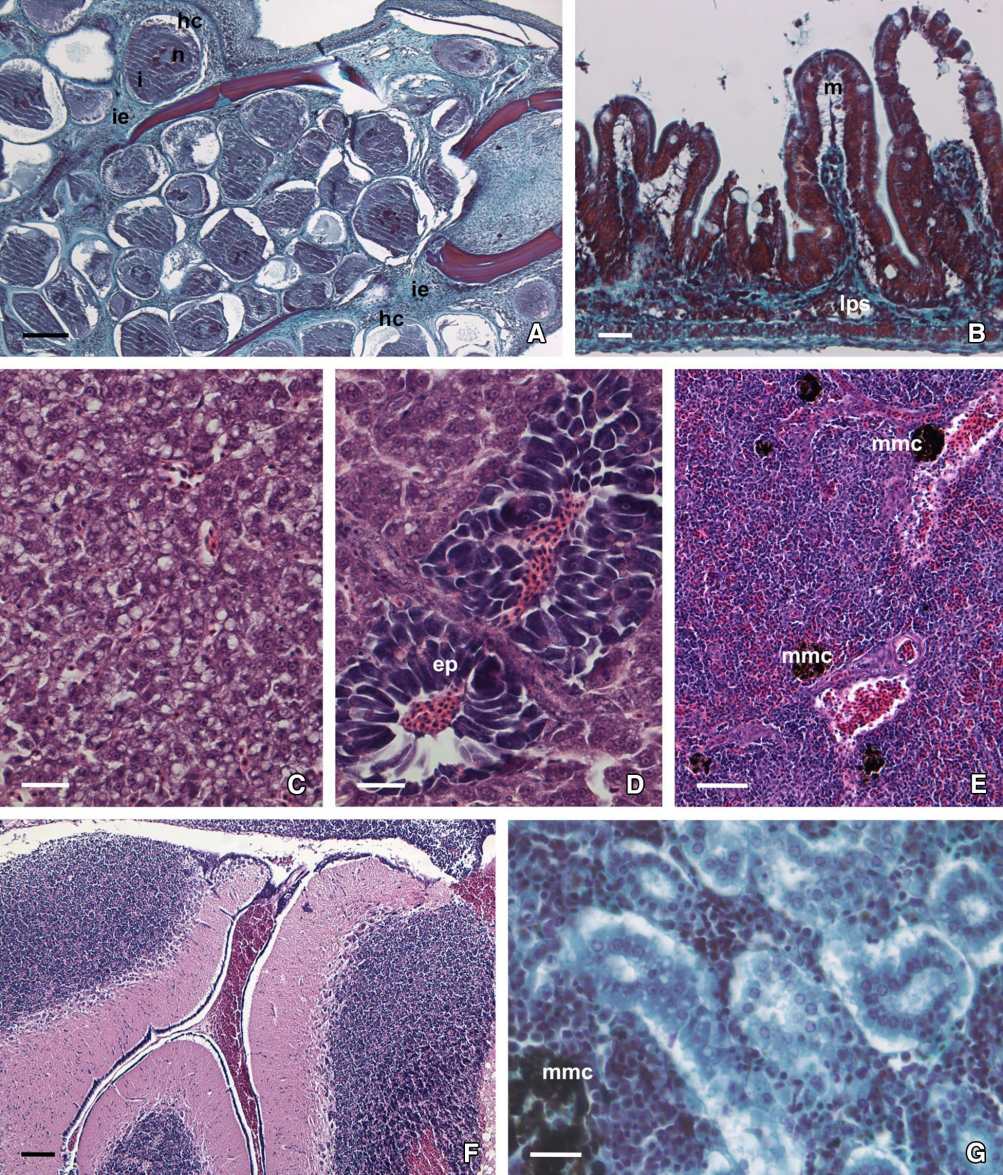
**Histopathology of lymphocystis disease in gilthead seabream juveniles. A** Caudal fin section with lymphocysts in several developmental stages (haematoxylin-V.O.F.); hyaline capsule (hc), nucleus (n); cytoplasmic inclusion (i); inflammatory epithelioid cells (i.e.). **B** Cross-section of the intestinal villi showing oedema in the mucosa (m) and inflammatory reaction in the lamina propria/submucosa (lps). The mucosal epithelium appears hyperchromatic (haematoxylin-V.O.F.). **C** Histological section of liver showing hepatocytes with cytoplasmic vacuolization and loss of polygonal shape (HE). **D** Pancreatic acinar cells (ep) exhibiting retraction and disruption of their structure (HE). **E** Spleen section showing numerous melanomacrophage centres (mmc) (HE). **F** Histological section of brain showing haemorrhagic ventricles (HE). **G** Histological section of kidney showing hyaline necrosis and vacuolization in the epithelial cells of renal tubules (haematoxylin-V.O.F.). Scale bars: (**A**) 200 µm; (**B**–**D**) 50 µm; (**E**, **F**) 100 µm; (**G**) 20 µm.

In all the LC-diseased fish analysed, clusters of typical hypertrophied fibroblasts (lymphocysts), exhibiting dark inclusions within the cytoplasm and enclosed by a hyaline capsule, were observed in the dermis of the caudal fin where they were surrounded by an abundance of inflammatory epithelioid cells (Figure 4A). Regarding internal organs, no lymphocysts were detected, but different types of histological alterations were observed depending on the organ analysed. The intestinal villi appeared dilated, and oedematous separation of the mucosa and inflammation of the submucosa layer were evident (Figure 4B). Hepatocytes showed altered shape, signs of strong vacuolization and increased cytoplasmic basophilia (Figure 4C), with some areas of hyaline necrosis (pyknotic nuclei) in the hepatic parenchyma and noticeable MMC. Several exocrine pancreatic cells showed some retraction and disruption of typical acinar structure (Figure 4D). An increase in the number of MMC was also observed in the splenic parenchyma (Figure 4E). Interestingly, brain ventricles appeared haemorrhagic in diseased specimens (Figure 4F). Finally, in the proximal kidney, renal tubules were occluded and their epithelial cells showed strong hyaline degeneration, disorganization and noticeable vacuolization, as well as nuclear changes observed in pyknotic cells. Moreover, as in the liver and spleen, MMC number increased in the kidney of diseased fish (Figure 4G).

**Figure 5:**
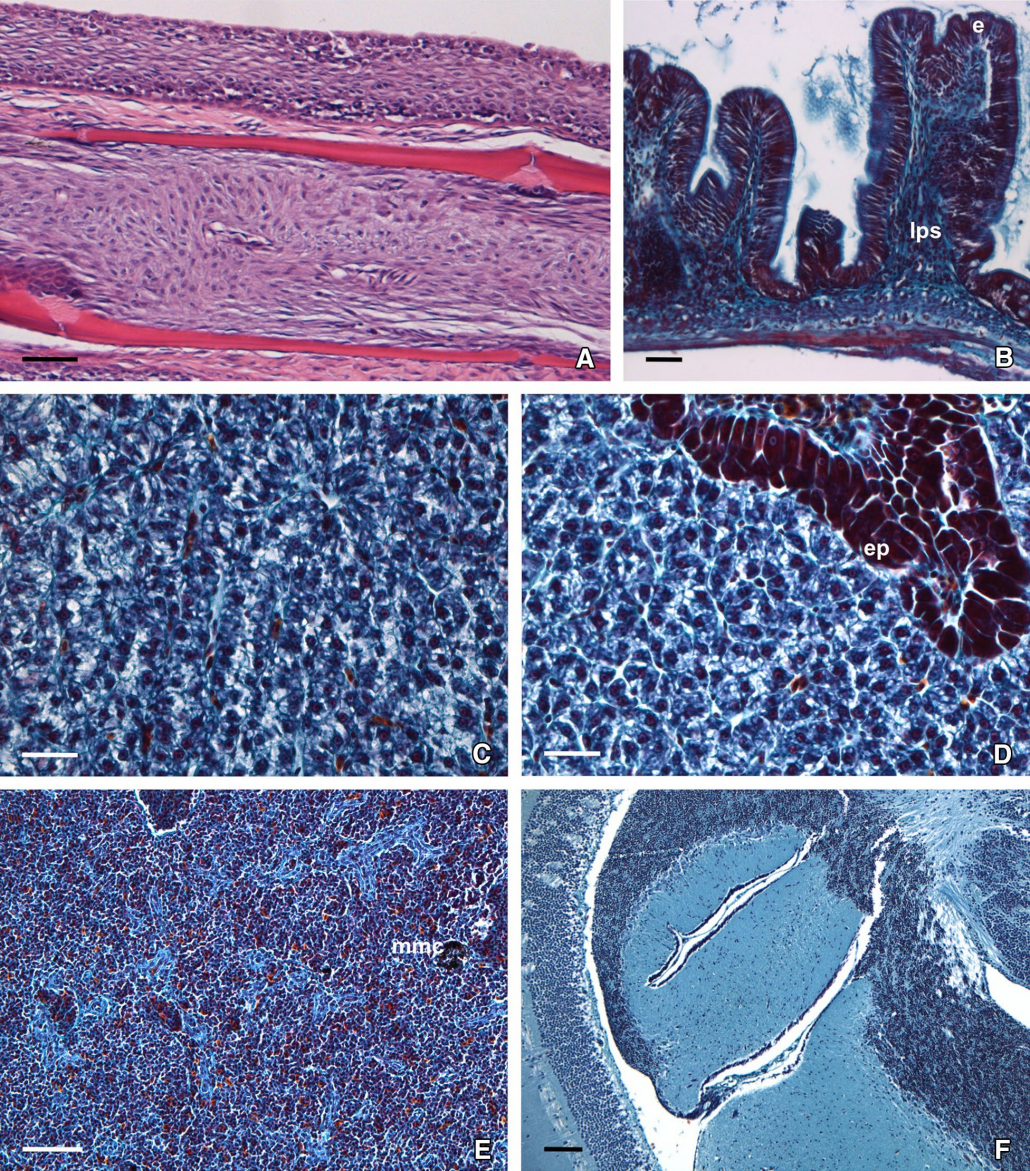
**Histopathology of gilthead seabream recovered from lymphocystis disease. A** Caudal fin section showing a normal structure (HE). **B** Cross-section of the intestinal villi showing hyperchromatic epithelium (e) and inflammation in lamina propria/submucosa (lps) (haematoxylin-V.O.F.). **C** Basophilic polygonal-shaped hepatocytes in liver section (haematoxylin-V.O.F.). **D** Portion of exocrine pancreas (ep) showing basophilic pancreatic acini (haematoxylin-V.O.F.). **E** Spleen section showing a few melanomacrophage centres (mmc) in the parenchyma (haematoxylin-V.O.F.). **F** Histological section of brain with empty cerebral ventricles (HE). Scale bars: (**A**–**D**) 50 µm; (**E**, **F**) 100 µm.

**Figure 6:**
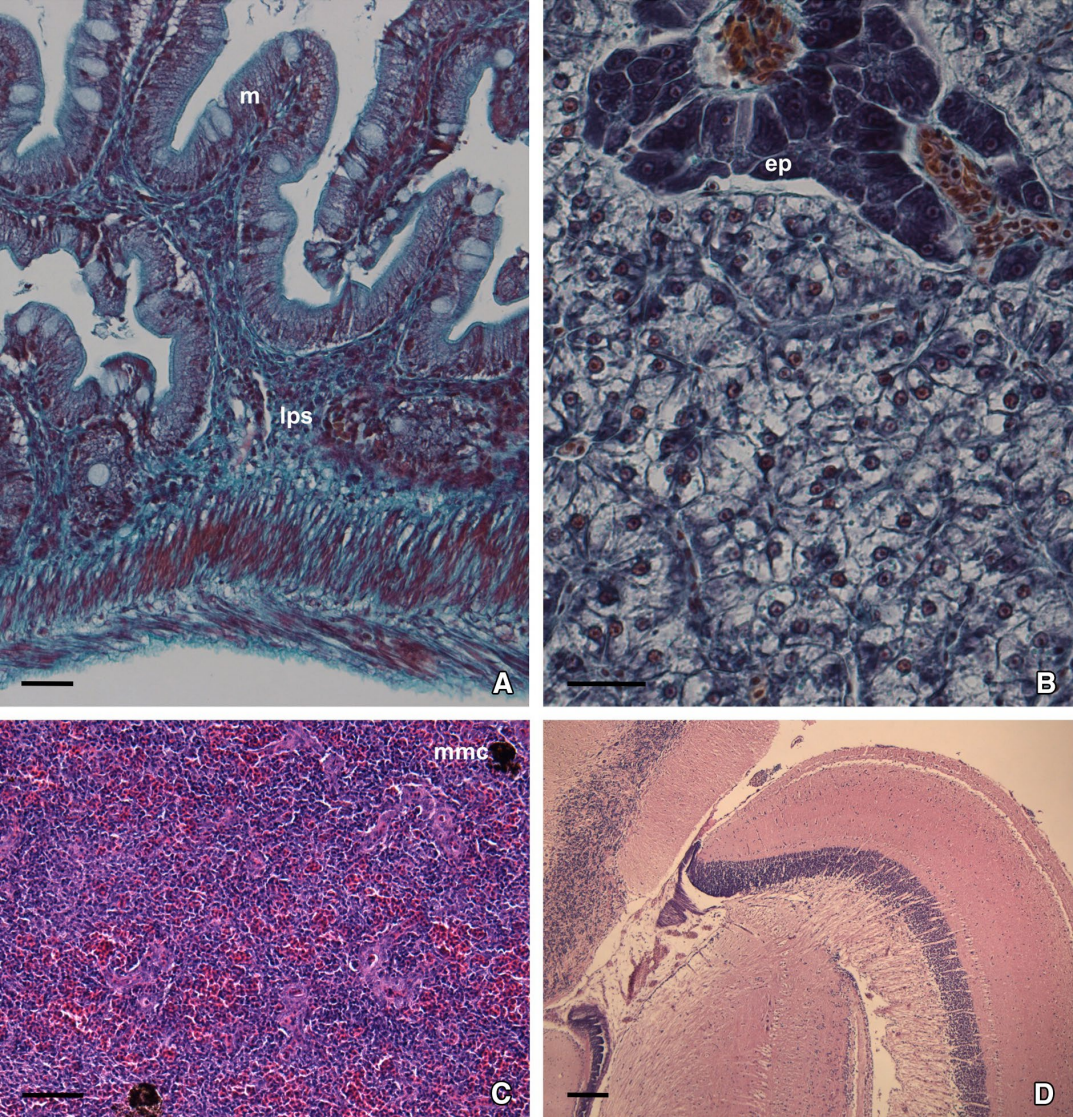
**Histology of asymptomatic gilthead seabream. A** Intestine section showing normal structure of the mucosa (m) and the lamina propria/submucosa (lps) (HE). **B** Histological section of kidney showing hepatocytes with polygonal shape, clear cytoplasm and nucleus in the periphery. Portion of exocrine pancreas (ep) with basophilic pancreocytes distributed in acini (haematoxylin-V.O.F.). **C** Histological section of spleen with a few melanomacrophage centres (mmc) (HE). **D** Histological section of brain (HE). Scale bars: (**A**, **B**) 50 µm; (**C**) 100 µm; (**D**) 200 µm.

When gilthead seabream specimens had recovered from LCD, most organs and tissues showed a normal structure and cellular pattern (Figure 5), resembling those observed in fish from the asymptomatic group (Figure 6) or in healthy gilthead seabream specimens from similar studies. Indeed, the caudal fin recovered its normal structure without a trace of lymphocysts (Figure 5A). Oedematous signs in the intestinal mucosa disappeared, although some dilatation of the intestinal brush border was still visible (see Figures 5B vs 6A). Hepatocytes showed their characteristic polygonal shape, although their cytoplasm appeared slightly basophilic with small signs of steatosis (see Figures 5C vs 6B). In fish from both the recovered and the asymptomatic groups, a normal architecture of the exocrine pancreas was observed (Figures 5D, 6B), and brain ventricles appeared without haemorrhagic focus (Figures 5E, 6C). In the spleen, the number of MMC in the recovered fish was reduced in comparison to diseased fish, and similar to that observed in asymptomatic animals (Figures 5F, 6D). Additionally, in the recovered fish, renal tissue showed signs of recovery and a small number of MMC was seen and the necrosis focus disappeared.

## Discussion

The pathognomonic signs of LCD include the appearance of small pearl-like nodules on the skin and fins that are usually grouped in clusters, papillomatous in appearance, and can cover the entire body surface of the fish [8]. These nodules consist of LCDV-infected hypertrophied dermal fibroblasts (up to 1 mm in diameter), named lymphocysts or lymphocystis cells [11, 21, 22]. LCDV is considered a dermatropic virus [8]; however, in some fish species, lymphocysts have also been observed in the mesenteries, peritoneum, and several internal organs, which could indicate that the infection can become systemic under certain conditions [4, 7, 23, 24]. Moreover, using sensitive immunological and molecular diagnostic methods, LCDV has been detected in different organs of fish without internal lesions [10, 13, 15, 16, 25]. These findings suggest a systemic condition for LCD although it has not been shown if viruses detected in different organs proceed from productive infections or are actually the result of an underlying viraemia.

In the present study, lymphocystis cells were exclusively observed in the dermis of the skin and fins of diseased juvenile gilthead seabream. Nevertheless, viral genomes were detected by qPCR in all of the organs analysed. Viral gene expression was also detected in all the samples, with the highest relative expression values recorded in the caudal fin, followed by those in the kidney and brain. Accordingly, the highest viral loads were detected in the fins, and the amount of viral genomes in the kidney and brain were significantly (*p* < 0.01) higher than in other internal organs analysed. These results support that LCDV establishes a systemic infection in gilthead seabream, similar to infections reported for other iridoviruses, such as ranaviruses and megalocytiviruses [26, 27]. Recent studies carried out in Japanese flounder and turbot have shown that LCDV genome copy numbers increased in all organs analysed during the course of experimental infections, and that the extensive range of viral target tissues is, at least partially, the result of the wide distribution of the LCDV-C receptor [28, 29]. Whether this receptor, a membrane protein of 27.8 kDa first identify in FG cells [30, 31], is present in gilthead seabream cells, and also a receptor for LCDV-Sa attachment, needs to be investigated.

Viral MCP transcripts were detected by ISH in order to identify susceptible cells supporting LCDV productive infection. As expected, LCDV expression was observed on lymphocysts located on the caudal fin but also in some cells in the surrounding connective tissue. Viral transcripts were also detected in hepatocytes, and in cells of the splenic pulp, the kidney interstitium, and the brain granular layer. This distribution of viral mRNA is similar to results of previous work that detected viral genomes and antigens in several organs of juvenile gilthead seabream [14]. In the present study, it was not possible to determine which cell type contained viral transcripts in the intestine. Nevertheless, Cano et al. [14] detected LCDV-positive cells in the connective tissue of the lamina propia. Furthermore, other authors also described the detection of LCDV genomes and/or antigens in the gill lamella of LC-diseased Japanese flounder, black rockfish, and gilthead seabream [13–15]. Together, these results support a broad range tissue tropism for LCDV, similar to that established for megalocytiviruses, which were described to be mesotheliotropic [32, 33].

On the basis of the results obtained, the permissive cells for LCDV replication seem to be fibroblasts, hepatocytes, and cells of the mononuclear phagocyte system, as previously suggested [14, 34]. The LCDV-C receptor has been detected in the membrane of a small portion of turbot peripheral leucocytes, which could indicate that they are susceptible to LCDV infection, resulting in LCDV spreading to different host tissues via the bloodstream [28]. In the gilthead seabream brain, viral transcripts were detected in cells of the granular layer, which suggests that microglial cells or infiltrating macrophages may be susceptible to LCDV, although neurons cannot be ruled out as a susceptible cell type. Further immunocytochemical studies should be carried out to identify LCDV-infected cells in brain, using, for example, OX-42 or FL.1 antibodies that recognize monocyte-derived cells in fish [35].

LCDV was detected at low levels in all of the organs analysed from asymptomatic and recovered fish. In addition, these organs support viral gene expression, indicating that the fish are subclinically infected by LCDV, and that this infection is also systemic. In asymptomatic fish, the highest relative viral expression value was recorded in the caudal fin, whereas the brain seems to be the main organ that supports viral expression in the recovered fish. As previously suggested by other authors [10, 14], LCDV establishes a systemic and persistent infection in gilthead seabream juveniles, which may extend for at least 2 months after disappearance of clinical signs.

Subclinically infected fish may be essential for LCD epizootiology. Thus, asymptomatic fish have been considered responsible for LCD outbreaks that appear in aquaculture facilities under stressful rearing conditions [36–39]. These conditions might stimulate virus replication and the consequent development of symptoms [40]. Moreover, it is assumed that fish can recover from LCD and develop acquired immunity [24, 41]. Nevertheless, recovered fish are persistently infected and, consequently, may be LCDV-carriers that could transmit the virus to naïve fish.

Histopathological studies carried out in LC-diseased fish have been focused on the description of lymphocystis cells, with few reports dealing with histological observations of internal organs, except when lymphocysts were also present [4, 9, 11, 12]. In the present study, LC-diseased gilthead seabream specimens showed lymphocystis cells only in the dermis of the caudal fin, with histological characteristics resembling those previously described in this fish species [9]. Histological alterations of different severities were also observed in all of the organs analysed, including necrotic changes in the liver and kidney, inflammatory response in the intestine submucosa, and intraventricular haemorrhage. Necrotic changes in the epithelium of the proximal renal tubules were the only histological alterations described so far in gilthead seabream juveniles affected by LCD [14, 42]. Nevertheless, histological damages similar to those observed in the present study in the liver, kidney or intestine, have also been described in LC-diseased snakeskin gourami (*Trichogaster pectoralis*) and kelp bass (*Epinephelus moara*) [22, 43]. These histopathological changes can be directly related to viral replication, as in the case of the liver, where hepatocytes are actually infected by LCDV (as demonstrated by ISH in the present study or by observation of viral particles by TEM in kelp bass), while in other cases an indirect relation could be proposed. Thus, epithelial necrosis of renal tubules has been associated with substances produced by infected cells in the interstitial tissue, or, alternatively, with severe alterations in osmoregulation resulting from multiple skin lesions [14, 42]. In addition, an increase in the number of MMC was observed in the liver, spleen, and kidney, which could be associated with a cellular response to viral infection [44]. The proliferation of epithelioid cells around lymphocysts has also been described as an immune response against LCDV [4, 12, 45]. Finally, in recovered fish, most organs and tissues showed normal histological features, indicating that histopathological alterations associated with LCD are reversible.

In conclusion, the results demonstrate that LCDV infection is a systemic condition in gilthead seabream, even for subclinical infections, where several organs seem to be primary or secondary targets for virus replication. LCDV has a broad range tissue tropism. In addition to dermis fibroblast that become lymphocysts after LCDV infection, cells from the liver, spleen, kidney, intestine, and brain could support a productive viral infection. The permissive cells for LCDV replication seem to be fibroblasts, hepatocytes and cells of the mononuclear phagocyte system.
